# Sex-Specific Proteomic Changes Induced by Genetic Deletion of Fibroblast Growth Factor 14 (FGF14), a Regulator of Neuronal Ion Channels

**DOI:** 10.3390/proteomes7010005

**Published:** 2019-01-23

**Authors:** Mark L. Sowers, Jessica Di Re, Paul A. Wadsworth, Alexander S. Shavkunov, Cheryl Lichti, Kangling Zhang, Fernanda Laezza

**Affiliations:** 1 UTMB MD/PhD Combined Degree Program, University of Texas Medical Branch, Galveston, TX 77555, USA; mlsowers@utmb.edu (M.L.S.); pawadswo@utmb.edu (P.A.W.); 2 Department of Pharmacology and Toxicology, University of Texas Medical Branch, Galveston, TX 77555, USA; jedire@utmb.edu (J.D.R.); asshavku@utmb.edu (A.S.S.); clichti@wustl.edu (C.L.); kazhang@utmb.edu (K.Z.); 3 Neuroscience Graduate Program, University of Texas Medical Branch, Galveston, TX 77555, USA; 4 Biochemistry and Molecular Biology Graduate Program, University of Texas Medical Branch, Galveston, TX 77555, USA

**Keywords:** mass spectroscopy, bioinformatics, FGF14, voltage gated channels, schizophrenia, autism, Alzheimer’s Disease, sex-specific differences, synaptic plasticity, cognitive impairment, excitatory/inhibitory tone

## Abstract

Fibroblast growth factor 14 (FGF14) is a member of the intracellular FGFs, which is a group of proteins involved in neuronal ion channel regulation and synaptic transmission. We previously demonstrated that male *Fgf14^−/−^* mice recapitulate the salient endophenotypes of synaptic dysfunction and behaviors that are associated with schizophrenia (SZ). As the underlying etiology of SZ and its sex-specific onset remain elusive, the *Fgf14^−/−^* model may provide a valuable tool to interrogate pathways related to disease mechanisms. Here, we performed label-free quantitative proteomics to identify enriched pathways in both male and female hippocampi from *Fgf14^+/+^* and *Fgf14^−/−^* mice. We discovered that all of the differentially expressed proteins measured in *Fgf14^−/−^* animals, relative to their same-sex wildtype counterparts, are associated with SZ based on genome-wide association data. In addition, measured changes in the proteome were predominantly sex-specific, with the male *Fgf14^−/−^* mice distinctly enriched for pathways associated with neuropsychiatric disorders. In the male Fgf14^−/−^ mouse, we found molecular characteristics that, in part, may explain a previously described neurotransmission and behavioral phenotype. This includes decreased levels of ALDH1A1 and protein kinase A (PRKAR2B). ALDH1A1 has been shown to mediate an alternative pathway for gamma-aminobutyric acid (GABA) synthesis, while PRKAR2B is essential for dopamine 2 receptor signaling, which is the basis of current antipsychotics. Collectively, our results provide new insights in the role of FGF14 and support the use of the *Fgf14^−/−^* mouse as a useful preclinical model of SZ for generating hypotheses on disease mechanisms, sex-specific manifestation, and therapy.

## 1. Introduction

Originally identified as the genetic locus of missense mutations leading to spinocerebellar ataxia type 27 [1,2,3,4,5,6,7], fibroblast growth factor 14 (FGF14) is an emerging risk factor for neuropsychiatric disorders [8]. Unlike canonical secreted FGFs, which act through the activation of FGF receptor signaling, FGF14 is retained intracellularly, where it has been shown to regulate ion channel function [9,10,11,12,13]. Much evidence indicates that FGF14 within neurons binds directly to and regulates the voltage-gated sodium (Nav) channel, targeting the axonal initial segment (AIS) and biophysical properties [9,10,11,12,13,14,15,16,17,18,19,20,21]. Other reported functions of FGF14 suggest a much more complex role within the brain, including the regulation of presynaptic glutamate and gamma-aminobutyric acid (GABA) release, and calcium signaling [18,19,20,21]. Studies focused on signaling pathways demonstrated that FGF14 is also a hub for regulatory kinases [11,22], including glycogen synthase kinase 3 [15], which is an enzyme that is linked to depression, bipolar disorder, and schizophrenia (SZ) [8,23,24,25].

Given the variety of key cellular functions associated with FGF14, it is not surprising that the deletion of the gene results in disrupted function and behavior associated with complex brain disorders. Recent studies have shown that male mice lacking *Fgf14* (*Fgf14^−/−^)* recapitulate key features of SZ endophenotypes. Namely, male *Fgf14^−/−^* mice present with the loss of parvalbumin positive GABAergic interneurons in the hippocampus, disrupted gamma frequency, and reduced working memory, all of which are hallmarks of cognitive impairment in SZ animal models and post-mortem studies [21,26]. Concomitant changes in these mice are found at the glutamatergic synapses with reduced presynaptic release and long-term potentiation [20,27], which may be the common underlying pathology of SZ and other neurodevelopmental disorders [28]. Additional evidence of disease endophenotypes is brought by studies reporting disrupted adult neurogenesis in the dentate gyrus (DG) of *Fgf14^−/−^* mice that is consistent with an immature dentate gyrus [21,29] and is another hallmark of SZ and other neuropsychiatric disorders [30].

In addition to reduced working memory, male *Fgf14^−/−^* mice exhibit behavioral deficits that align with disrupted dopamine signaling, including altered aggressive and reproductive behavior, and blunt response to cocaine and methamphetamine [26,31].

Taken together, these findings indicate that the male *Fgf14^−/−^* mouse recapitulates the endophenotypes of SZ, including changes in GABA and glutamatergic synaptic signaling, leading to perturbations of the excitatory/inhibitory (E/I) tone of the brain [32,33,34,35,36], impaired neurogenesis in the DG, and disruption of dopamine signaling, which are all functional nodes in SZ pathophysiology. 

Although many lines of evidence converge to suggest that male *Fgf14^−/−^* mice are useful animals for the study of SZ, little is known about how these complex phenotypes develop, how they relate to other neurodevelopmental diseases, or whether sex-specific differences exist in female *Fgf14^−/−^* animals.

We chose to investigate this potentially useful animal model to gain further insight into the etiology of SZ and related disorders. We performed label-free proteomic mass spectrometry and a variety of bioinformatic approaches on isolated hippocampi from male and female wild-type (WT) and *Fgf14^−/−^* mice to determine the molecular pathways disrupted in this model. As a result, we found evidence that this animal model recapitulates the molecular aspects found in patients afflicted with SZ. Our results will aid in the generation of new hypotheses about neuropsychiatric diseases, and are expected to elucidate several gender-specific differences in the etiology of SZ, such as the age of diagnosis, symptom clustering, premorbid function, treatment response, and prognosis [37,38,39,40,41].

## 2. Materials and Methods 

### 2.1. Hippocampal Tissue Preparation

*Fgf14^−/−^* and *Fgf14^+/+^* male and female mice are maintained on an inbred C57/BL6J background with greater than 10 generations of backcrossing to C57/BL6J. Animals were bred in the University of Texas Medical Branch animal care facility: either heterozygous *Fgf14^+/−^* males and females or, in a few cases, homozygotes (*Fgf14^−/−^* males with *Fgf14^+/−^* females); *Fgf14^+/+^* WT mice served as control. Both male and female mice were used in this study at four to six months of age, unless otherwise stated. The University of Texas Medical Branch operates in compliance with the United States Department of Agriculture Animal Welfare Act, the Guide for the Care and Use of Laboratory Animals, and Institutional Animal Care and Use Committee approved protocols (0904029C). Mice were housed, *n* ≤ 5 per cage, and kept under a 12-h light/12-h dark cycle with sterile food and water *ad libitum*. All of the genotypes described were confirmed by genotyping of the progeny using DNA extraction and PCR amplification following established protocols or conducted at Transnetyx Inc. (Cordova, TN, USA). 

Both hippocampi were dissected from each mouse brain of *Fgf14^−/−^* and *Fgf14^+/+^* male and female adult mice. A total of three biological replicates were in each group. Biological replicates were combined to maximize the amount of total protein. Protein extraction was done on these combined samples and analyzed three times for a total of three technical replicates. Tissue was homogenized in RIPA buffer (Thermo Fisher Scientific, Rockford, IL, 25 mM of TrisHCl pH 7.6, 150 mM of NaCl, 1% NP-40, 1% sodium deoxycholate, 0.1% SDS) containing Halt protease and phosphatase EDTA-free inhibitor cocktail (Thermo Fisher Scientific, Rockford, IL, USA) and one mM of phenylmethylsulfonyl fluoride. Mechanical homogenization was performed using Polytron™ PT 10/35 GT Homogenizer (Kinematica, Bohemia, NY, USA), 20 s × three pulses, at 10,000 rpm. After homogenization, Pierce universal nuclease (Thermo Fisher Scientific, Rockford, IL) was added to samples (25 units per one mL of tissue lysate) and incubated on ice for 30 min. Protein concentration was determined using a BCA Protein Assay Kit (Pierce). Then, 100 μg aliquots of total protein were reduced and alkylated. 5 μL of 200 mM of tris (2-carboxyethyl) phosphine (TCEP) buffered with 50 mM of triethylammonium bicarbonate (TEAB) were added to each sample (final TCEP concentration: 10 mM) and incubated at 55 °C for 1 h. 5 μL of 375 mM of iodoacetamide (buffered with 50 mM of TEAB) were added and incubated in the dark for 30 min. Proteins were precipitated in four volumes (440 μL) of ice-cold acetone overnight at −20 °C. Samples were centrifuged at 10,000× *g* for 30 min (4 °C), after which the supernatants were removed and discarded. Protein pellets were delipidated and incubated in one mL of ice-cold tri-n-butylphosphate/acetone/methanol (1:12:1 by volume), followed by centrifugation (Eppendorf 5415D, Hamburg, Germany) at 2800× *g* for 15 min at 4 °C, and sequential incubations in ice-cold tri-n-butyl phosphate, acetone, and methanol, for 15 min each [42]. Pellets were air-dried and resuspended in 12.5 μL of eight M of urea. Trypsin (4 μg in 87.5 μL of TEAB buffer) was added, and the samples were incubated for 24 h at 37 °C. A final sample clean-up and removal of urea were performed using Mark C18 Sep-Pak^®^ Vac 1cc cartridges (Waters, Milford, MA, USA) attached to a vacuum manifold. Cartridges were pre-equilibrated with 3 × 1 mL of acetonitrile and washed with 3 × 1 mL of 0.25% trifluoroacetic acid (flow rate ~ 2 mL/min); digested samples were loaded onto the cartridges after adding trifluoroacetic acid to 1% final concentration, washed with 4 × 1 mL of 0.25% trifluoroacetic acid, eluted in one mL of 80% acetonitrile/0.1% formic acid, and dried in the CentriVamp Concentrator (Labconco, Kansas City, MO, USA).

### 2.2. Mass Spectrometry and Chromatography

Chromatographic separation and mass spectrometric analysis were performed with a nano-LC chromatography system (Easy-nLC 1000, Thermo Scientific) coupled online to a hybrid linear ion trap-Orbitrap mass spectrometer (Orbitrap Elite, Thermo Scientific) through a Nano-Flex II nanospray ion source (Thermo Scientific). Mobile phases were 0.1% formic acid in water (A) and 0.1% formic acid in acetonitrile (ACN, B). After equilibrating the column in 95% solvent A and 5% solvent B, the samples (5 μL in 5% *v*/*v* ACN/0.1% (*v*/*v*) formic acid in water, corresponding to 1 μg of tissue protein digest) were injected onto a trap column (C18, 100 μm ID × 2 cm) and subsequently eluted (250 nL/min) by gradient elution onto a C18 column (10 cm × 75 μm ID, 15 μm tip, ProteoPep II, 5 μm, 300 Å, New Objective). The gradient was as follows: isocratic flow at 5% Solvent B for 5 min, 5% to 35% Solvent B for 89 min, and 35% to 95% Buffer B for 16 min followed by isocratic flow at 95% Buffer B for 10 min. 

All of the LC-MS/MS data were acquired using XCalibur, version 2.7 SP1 (Thermo Fisher Scientific). The survey scans (*m*/*z* 350–1650) (MS) were acquired in the Orbitrap at 60,000 resolution (at *m*/*z* = 400) in profile mode, followed by top five higher energy collisional dissociation (HCD) fragmentation centroid MS/MS spectra, acquired at 15 K resolution in data-dependent analyses (DDA) mode. The automatic gain control targets for the Orbitrap were 1 × 10^6^ for the MS scans and 5 × 10^4^ for MS/MS scans. The maximum injection times for the MS1 and MS/MS scans in the Orbitrap were both 500 ms. For MS/MS acquisition, the following settings were used: parent threshold = 10,000; isolation width = 4.0 Da; normalized collision energy = 30%; and activation time = 10 ms. zMonoisotopic precursor selection, charge-state screening, and charge-state rejection were enabled, with the rejection of singly charged and unassigned charge states. Dynamic exclusion was used to remove selected precursor ions (±10 ppm) for 90 s after MS/MS acquisition. A repeat count of one, and a maximum exclusion list size of 500, were used. The following ion source parameters were used: capillary temperature 275 °C, source voltage 2.1 kV, source current 100 μA, and S-lens RF level 40%. Each sample was analyzed in triplicate, and the order of runs was block-randomized.

### 2.3. Quantification of Peptides and Proteins

Maxquant version 1.6.1.0 was used to process raw files [43,44]. Default settings were used unless otherwise specified. Briefly, peptide spectrum match and protein false discovery rate (FDR) were set to 1% and a minimum of one unique peptide for identification. Fixed modifications were set to carbamidomethyl for cysteine, and variable modifications were set to methionine oxidation and N-terminal acetylation. Matches between runs were enabled with a default match time window of 0.7 min and alignment window of 20 min. The mouse Uniprot reference proteome was downloaded on 18 September 2018, last updated 28 July 2018, with canonical and isoform sequences. For label-free quantification (LFQ), MS2 was required, while a minimum of one peptide was required for quantification across samples, including both razor and unique peptides. 

### 2.4. Statistical Analysis

Statistical analysis was performed with Perseus 1.6.0.7 [45]. LFQ intensity values were log_2_ transformed to render the data normally distributed. Proteins identified by site, reverse, and potential contaminants were filtered prior to analysis. Proteins with missing values in any sample, including replicates, were filtered. Differentially expressed proteins were determined using a moderated *t* test statistic with the FDR controlled at 5% and the s0 parameter set to 0.1. Multiple test correction was done using a permutation-based randomization procedure where values are randomly shuffled to generate a “null” distribution to estimate the random type one error, or false detection rate, with 250 randomizations. This is the preferred procedure in Perseus. 

## 3. Results

**Figure 1:**
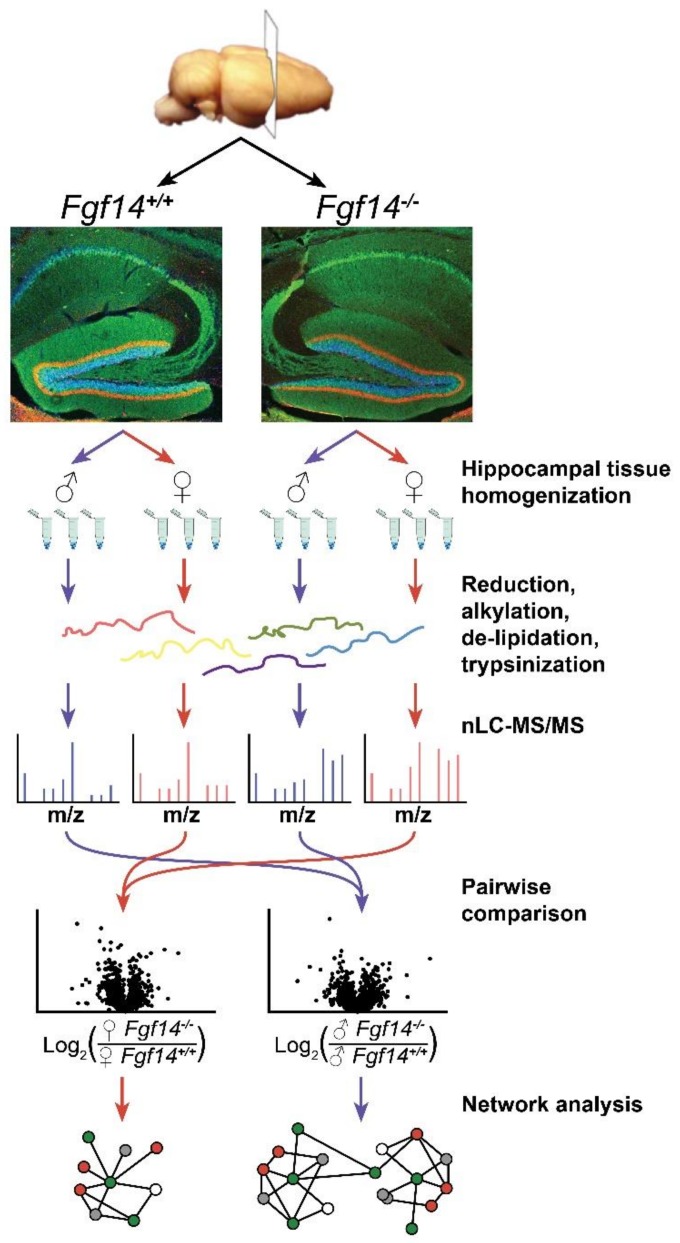
Overview of label-free proteomics workflow and analysis. Workflow outlining experimental procedures and LC-MS/MS data acquisition for the analysis of hippocampal brain tissue [50,51,52] from male and female *fgf14^+/+^* and *Fgf14^−/−^* mice, as detailed in the text. Representative confocal images of triple staining of the entire hippocampus from *Fgf14^+/+^* (**left**) and *Fgf14^−/−^* (**right**) mice representing calbindin (green), calretinin (red), and Topro-3 nuclear staining (blue) at low magnification of the dentate gyrus (DG).

To efficiently detect specific changes in the cellular proteome, it is important to limit the biological complexity of the subject of study. Whole brain proteome analysis is likely to miss or downplay prominent changes of protein expression in particular brain regions. Therefore, the proteomic analysis of isolated brain structures is preferred. Previous studies, including our own, have largely focused on the role of FGF14 in the hippocampus. The hippocampus is part of the limbic system, which is critically involved in cognition and a primary site of FGF14 expression [13,20,31]. FGF14 knockout causes pronounced changes in the synaptic transmission [20,21] and cellular composition [21,29] of the hippocampus, which correlate with changes in electrophysiology and behavioral deficits [20,27,31]. Given the documented role of hippocampal pathology in cognitive impairment in SZ [46,47,48,49], we hypothesize that these gross changes play a critical part in the development of SZ-related endophenotypes in *Fgf14^−/−^* mice. An additional advantage is that the hippocampus can be readily isolated from adjacent brain structures, which makes sample preparation more robust and reproducible. The workflow of our study is presented in Figure 1. 

**Figure 2:**
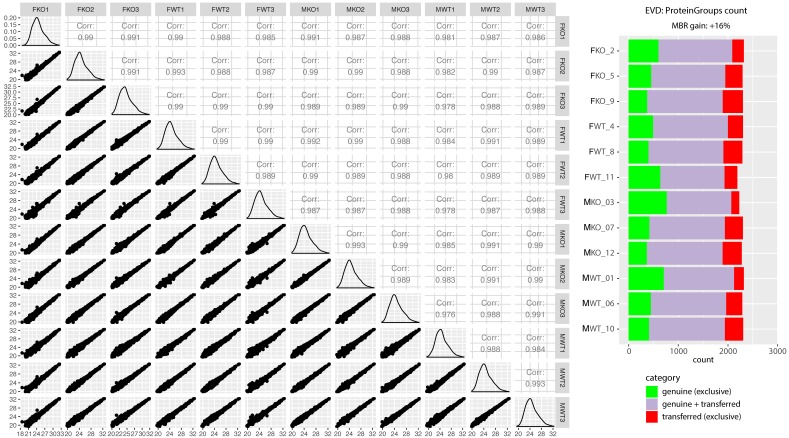
Quality control of label-free quantitative proteomics. The scatter matrix shows pairwise Pearson correlations between animal groups and their technical replicates, histograms of log_2_ label-free quantification (LFQ intensity) distributions, and their respective scatter plots. On the right is a quality control figure showing ~2300 proteins identified in each sample after applying match-between-runs (MBR) in Maxquant. Most proteins were identified by a combination of MS1 matching from other samples, where peptide identification was successful, as well as directly by MS2 (purple). A smaller subset of proteins could be identified exclusively by MS2 in a given run (green), and proteins identified only after the retention time and the *m/z* alignment of MS1 peaks in comparison to other runs resulted in a 16% gain of quantified proteins (red). The quality control figure was prepared using R programming language and Proteomics Quality Control (PTXQC) [53].

While having many advantages, primarily ease of use, label-free proteomics chromatography conditions must be standardized and assessed for reproducibility and overall data quality. As shown in Figure 2, the various samples and their technical replicates are highly reproducible after appropriate filtering (see methods). Furthermore, Maxquant quantifies protein intensity using MS2 spectra. However, if an MS2 spectra, which is needed for peptide sequencing/identification, is missing in one run due to the stochastic nature of data-dependent acquisition, Maxquant has the match between run (MBR) feature. MBR allows for quantification by imputing the estimated MS2 intensity by using the mass and retention time alignment of the corresponding MS1 peak [43,44]. 

### 3.1. Differentially Expressed Proteins in Fgf14^−/−^ Mice and their Implications

**Figure 3:**
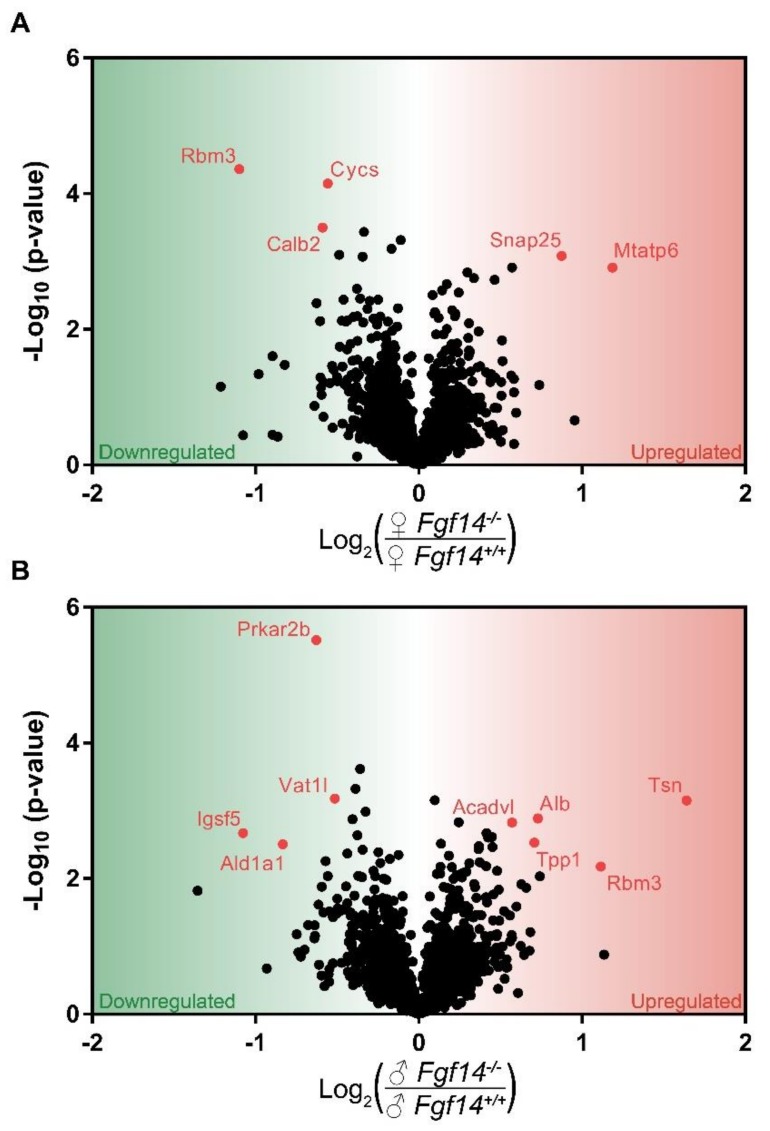
Volcano plot of mass spectrometry results. Proteins that are significantly upregulated in sex by genotype conditions: (**a**) shows the proteins significantly upregulated in female *Fgf14^−/−^* compared to *Fgf14^+/+^* mice; and (**b**) shows the proteins significantly upregulated in male *Fgf14^−/−^* compared to *Fgf14^+/+^* mice. The Y-axis represents negative log10 (*p*-value) based on the test statistic, and the X-axis shows proteins with a positive log_2_ fold change (FC) as upregulated (red), and negative values as downregulated (green) proteins in the *Fgf14^−/−^* mice, respectively.

After log transforming and filtering, we analyzed ~1500 proteins whose distribution was approximately normal across all of the samples. Then, we compared male and female *Fgf14^−/−^* mice to their respective wild-type counterparts using statistical analysis of microarrays, which is a moderated t-test statistic (Figure 3, Table S1). We chose to investigate both male and female homozygous knockouts of FGF14, as we had previously shown that male knockouts have SZ-like dysfunction, while female mice for this model had not been previously investigated. 

In the female *Fgf14^−/−^* mice, we found *Snap25* and *Mtatp6* upregulated. SNAP25 is part of the SNARE complex, which mediates neurotransmitter–vesicle fusion and controls receptor trafficking at post-synaptic sites of glutamatergic and GABAergic synapses [54]. It is unclear how the genetic deletion of FGF14 causes a change in SNAP25 expression, but previous proteomic studies have shown that FGF14 immunoprecipitates with SNAP25 [10]. Thus, the genetic deletion of FGF14 could lead to SNAP25 loss of function, which in animal models is considered a mechanism leading to SZ endophenotypes [55]. 

MTATP6, or ATP synthase/Complex V, has been associated with SZ as either decreased mRNA levels or as genetic polymorphisms [56,57]. Thus both SNAP25 and MTATP6, which have been shown either knocked down or decreased in association with SZ, are upregulated in female knockouts; this is a possible mechanism of resistance to the genetic deletion of FGF14. SNAP25 and MTATP6, while upregulated in female mice, were not differentially expressed in male mice after multiple hypothesis test correction. This suggests that the cognitive deficits seen in male *Fgf14^−/−^* mice may be a consequence of reduced energy production, while their female *Fgf14^−/−^* counterparts may be able to compensate. Despite the upregulation of these two proteins, we would expect some dysfunction in mitochondrial energy production as well as GABA-ergic signaling in the female knockout mice, as represented by decreased Calretinin (CalB2) and Cytochrome C (Cycs). 

CYCS is an essential component of oxidative phosphorylation that is a major source of energy, particularly in neurons. Mitochondrial dysfunction is also believed to be one of the potential risk factors of SZ [56]. CALB2 is a calcium-buffering protein that is predominantly expressed in calretinin positive interneurons, which is a subtype of cells expressed in the hippocampus [58]. This suggests that there may be a decrease in calretinin-positive interneurons in the hippocampus in female *Fgf14^−/−^* mice. This is in direct opposition to the increase in these interneurons and immature dentate gyrus that were previously reported in *Fgf14^−/−^* males [29].

Differentially expressed proteins were almost entirely different between female *Fgf14^−/−^* and male *Fgf14^−/−^* compared to their respective *Fgf14^+/+^* controls, with the exception of RBM3. RBM3 is a cold inducible protein that is believed to be protective against neurodegeneration and mediate structural plasticity [59]. While believed to aid in translation, RBM3 has also been reported as two alternatively spliced isoforms, with the variant lacking arginine more highly expressed in the dendritic spines of mature neurons [60]. Smart et al. also reported in the same study that both RBM3 isoforms are post-translationally modified. Thus, the difference in RBM3 expression between male and female *Fgf14^−/−^* mice could be due, in part, to the lack of quantitation of some peptides, since only unmodified peptides were quantified. While RBM3 was found to be upregulated in males, which is generally thought to be protective, it is unclear if this is due to the stress response, lack of post-translational modifications, or some combination of the two. Additionally, we found that RBM3 expression was lower in male versus female WT groups (Figure S1). This might suggest that females and males have either differential expression in the hippocampus; alternatively, again, sex-specific post-translational modifications could also play a role. Furthermore, differential expression may be a consequence of different dendritic morphology and branching [61]. There are known differences in male and female C57BL/6J mice, as RBM3 is enriched in dendritic spines. However, further targeted investigation would be needed in order to determine the effect of sex and FGF14 on RBM3 expression and post-translational modifications.

Although we focused our studies primarily on male knockouts (see Discussion), as they displayed the cognitive and synaptic functions of interest, we identified key differences between normal male and female hippocampi. Namely, most of the proteins that were differentially expressed were related to the “neuron part” cellular component of the gene ontology (GO) term (Figure S1). As mentioned previously, dendritic morphology has sex-specific differences. Estrogen may also play a role in the hippocampal neuronal spine shape and long-term potentiation [62]. Our results support that there are sex-dependent differences in proteins that are important for spine formation and dendrite morphology in the hippocampus. Proteins overexpressed in the females, relative to other WT mice that were male, were related to calcium signaling (CAMK2A) and calcium regulation (ANXA6). The former is most strongly implicated in the early phases of long-term potentiation [63]. Copine 6 was also upregulated, which is a calcium-binding protein that is believed to be responsible for translating calcium signals into morphological changes at the level of synaptic spines [64]. 

Interestingly, two differentially expressed proteins that were found downregulated in *Fgf14^−/−^* male mice than male WT, IGSF5 and VAT1L, were also found to be more abundantly expressed in the male WT than female WT. Not only do these proteins appear to be differentially regulated by loss FGF14 in only the male mice, they are more abundantly expressed in male WT than female. This suggests they may be of central importance in the male hippocampus. 

### 3.2. Differentially Expressed Proteins Highly Associated with Schizophrenia and/or Autism

**Figure 4:**
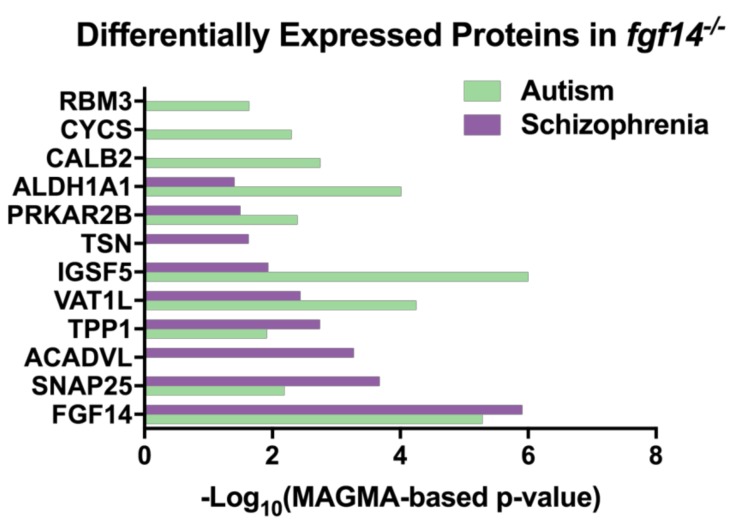
Differentially expressed proteins in *Fgf14^−/−^* mice are associated with autism and schizophrenia. The proteins that were identified in our study were found in an analysis of genome-wide association study (GWAS) data [65] (Table S10) using the MAGMA tool [66].

Interestingly, we discovered that in an analysis of various genome-wide association studies (GWAS), all of our differentially expressed proteins, with the exception of MTATP6, were identified to be statistically associated with either autism and/or SZ, suggesting that the *Fgf14* knockout mouse might be a valuable model for a wider range of neuropsychiatric and neurodevelopmental disorders. Protein level *p*-values were determined by Seyfried et al. using the MAGMA tool, which controls for various confounders to determine the *p*-values for each protein coding gene [65,66] (Figure 4, Table S2). MTATP6 may have been missing in this dataset due to being coded on the mitochondrial genome.

### 3.3. Central Role of ALDH1A1 and SNAP25 in Pathophysiology of Fgf14^−/−^ Mice

**Figure 5:**
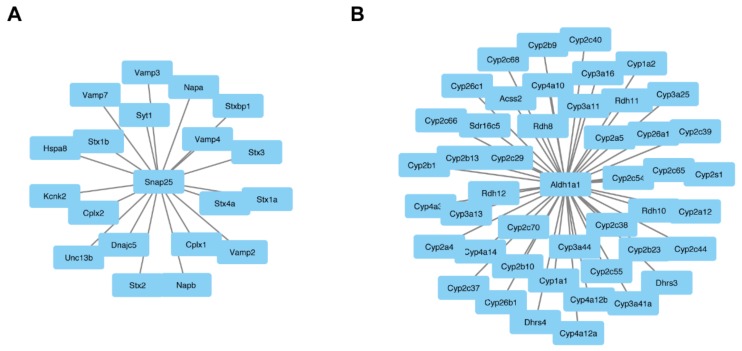
Central node proteins networks. OmicsNet was used to generate protein–protein networks with differentially expressed proteins and known experimental interactors. The networks that were created based on input gene names are shown for both the male and female *Fgf14^−/−^* mice. In the center are the input genes, and the connected genes are known interactors. This analysis identified *snap25* (**A**) and in female *Fgf14^−/−^* and a*ldh1a1* (**B**) male *Fgf14^−/−^* mice as central interactors.

Experimental protein–protein interaction networks were constructed with the differentially expressed proteins for males and females, separately, using OmicsNet, which identifies known interactors [67] (Figure 5). Interactions were based only on high-confidence STRING interactions with experimental evidence. The networks were imported into Cytoscape for visual purposes. Three-dimensional predicted protein–protein interaction networks were constructed with the differentially expressed proteins for males and females, separately (Figure 5)**.** The network construction did not generate any connections to other significant proteins other than *Snap25* and *Aldh1a1* for females and males, respectively. Although protein–protein interaction data are far from complete, this suggests that Snap25 and Aldh1a1 may be key players in the pathogenesis observed in *Fgf14^−/−^* mice and perhaps SZ and/or autism [68,69].

### 3.4. Hierarchical Clustering Reveals Subtype-Specific Clusters

**Figure 6:**
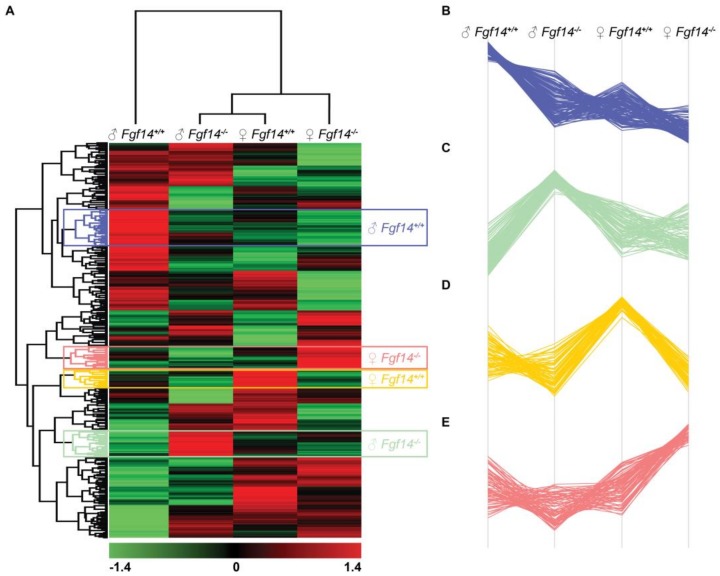
Hierarchical clustering, heatmap, and cluster analysis of differentially expressed proteins. (**A**) Heatmap of differentially expressed proteins in male and female *Fgf14^+/+^* and *Fgf14^−/−^* mice. LFQ intensities were averaged for technical replicates, and averages across animal groups were Z-scored prior to Euclidean distance-based hierarchical clustering with Perseus (**B**–**E**). Protein clusters specific to each animal group, male *Fgf14^+/+^*, female *Fgf14^−/−^*, female *Fgf14^+/+^*, and male *Fgf14^−/−^*, respectively.

**Figure 7:**
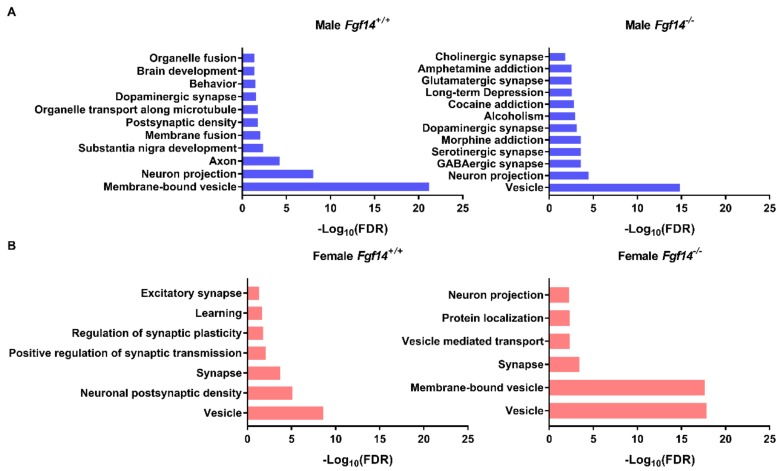
Protein–protein interaction and pathway enrichment for animal-specific clusters. (**a**) Enriched pathways and their adjusted *p*-values (FDR) were obtained from the STRING database after inputting cluster-specific gene names. Male *Fgf14^−/−^* display protein expression changes broadly associated with neurotransmitter-based synaptic activation, drug addiction, and alcoholism. This was unique to the male *Fgf14^−/−^* specific cluster. (**b**) Female *Fgf14^−/−^* mice display protein expression changes broadly associated with synaptic vesicles, synaptic transport, and protein localization; this is an important function of FGF14.

We performed the hierarchical clustering of quantified proteins and sample groups using Euclidean distance metric with average linkage as well as preprocessing with k-means for data reduction purposes, prior to the generation of the heatmap shown in Figure 6. Sample replicates were median-averaged, and the measured proteins were Z-score normalized across sample groups prior to clustering. Both Z-scoring and clustering were done in the Perseus bioinformatics suite (default clustering settings) [45]. Our analysis identified four protein clusters of interest, because they were upregulated in each of the respective animals. We submitted these group-specific clusters to the STRING protein–protein network database using only the highest confidence interactions based on all of the data types, and identified the positively enriched pathways for each animal-specific protein cluster (Figure 7, Table S3).

Of particular note are the enriched pathways in male *Fgf14^−/−^*mice, which includes alcoholism, drug addiction, and related pathologies. These pathways may explain the endophenotype of male *Fgf14^−/−^* mice. Furthermore, all of the animal groups had enriched terms related to vesicle, membrane-bound vesicle, or vesicle-mediated transport, likely indicating their important roles in the mouse hippocampus. This also suggests that both sex and the presence of FGF14 may affect different aspects of neurotransmission given that these terms are positively enriched in all of the protein clusters.

## 4. Discussion

Using a label-free proteomic approach and bioinformatics, we analyzed sex-specific differences in the hippocampi of *Fgf14^−/−^* mice relative to their sex-specific controls. Previous work has demonstrated that male *Fgf14^−/−^* mice present with cognitive deficits and changes in neuronal function that mimic the endophenotypes of SZ and other neuropsychiatric disorders [21,29]. However, the results presented in this study provide a biological context as to which specific pathways might be disrupted. Importantly, we found that many of the proteins differentially expressed in male *Fgf14^−/−^* mice have previously been linked to neuropsychiatric disorders with cognitive impairment, such as SZ and autism (Figure 4). In fact, a network analysis of proteomic data from the brains of Alzheimer’s disease (AD) patients has shown that synaptic transmission, synaptic membrane, and mitochondrion pathways are disrupted [65]. Perhaps this indicates a general mechanism for cognitive impairment that may be related to SZ, autism, and even the cognitive aspects of AD.

Importantly, many of the proteins with significantly altered expression in male *Fgf14^−/−^* mice, including ALDH1A1, PRKAR2B, and VAT1L, have previously been linked to SZ and other neuropsychiatric disorders within the domain of cognitive symptoms (Figure 3) [70,71,72,73]. These results also further support the role of FGF14 in synaptic signaling [18,21]. For example, it is known that *Fgf14^−/−^* male mice present with changes in GABA-ergic signaling in the hippocampus [18,21]. Our results here support that FGF14 may regulate the composition of GABA-ergic synapses both presynaptically and postsynaptically through SNAP25 (Figure 3) and synaptic function (Figure 7).

Alterations in the dopaminergic signaling of male *Fgf14^−/−^* mice may be due to changes in ALDH1A1. ALDH1A1 is not only an important enzyme for the breakdown of alcohol, it also defines a subpopulation of dopaminergic neurons in the rodent and human substantia nigra pars compacta, which are sensitive to α-synuclein cytotoxicity [71]. As shown in Liu et al., the deletion of ALDH1A1 exacerbates dopaminergic neurodegeneration in a mouse model of Parkinson’s disease. This effect may be mediated through changes in the E/I tone of the brain, as retinoic acid, which is synthesized by ALDH1A1, regulates synaptic scaling at glutamatergic synapses by regulating AMPA receptor trafficking [74]. Furthermore, ALDH1A1 is part of a highly conserved pathway that provides an alternative method of GABA synthesis through putrescine [70]. Disrupting this pathway through decreasing ALDH1A1 might cause the deprivation of alternative pathways to synthesize GABA, which could in turn reduce inhibitory transmission and disrupt the E/I tone. These findings support others who have shown that male *Fgf14^−/−^* mice exhibit changes in synaptic function as well as in their response to drugs of abuse, such as cocaine and methamphetamine [31].

Changes in dopaminergic and GABAergic signaling in male *Fgf14^−/−^* mice can also be attributed to a decreased level of protein kinase A (PRKAR2B). PRKAR2B has been linked to GABA receptor breakdown by the endothelial gene claudin-5 in the prefrontal cortex of patients with SZ [75], and may partially underlie the mechanism of action of several antipsychotics through the increase of GABA receptors [72,76]. PRKAR2B also plays a role in dopaminergic neuromodulation, although this has typically been shown in the nucleus accumbens for D2 receptor signaling and in neuronal firing in medium spiny neurons [75,77,78,79,80]. Probes that target the interface between FGF14 and the voltage-gated Na^+^ channel 1.6 (Nav1.6) have been shown to disrupt medium spiny neuron firing, which is a phenotype found in the same neuron subtype in male *Fgf14^−/−^* mice [14]. Therefore, it is plausible that protein kinase A (PKA) and FGF14 provide a regulatory mechanism of medium spiny neuron firing that contributes to maintaining dopaminergic tone in the nucleus accumbens.

Other proteins with altered expression in the male *Fgf14^−/−^* mice are members of pathways altered in neuropsychiatric disorders. Translin (TSN) is an RNA binding protein that regulates the dendritic trafficking of brain-derived neurotrophic factor (BDNF) [81]. BDNF is tied to synaptic transmission, plasticity, and homeostasis, and decreased serum levels of BDNF and mutations in the BDNF receptor, tyrosine receptor kinase B, have also been linked to SZ [82,83]. Although little is known about the effects of increased TSN [84], its altered expression in male *Fgf14^−/−^* mice, along with altered levels of expression of other SZ associated proteins, support additional findings that the male *Fgf14^−/−^* mouse model may be a new model of SZ and other disorders with a disrupted cognition component.

Overall, these findings support that *Fgf14^−/−^* male mice have several key features constituting an endophenotype of SZ [21]. As there are currently no pharmacotherapies for the treatment of the cognitive symptoms of SZ, this animal model may be a powerful tool in the discovery and testing of new disease treatments.

Of similar importance is the striking finding that the differentially expressed proteome of female *Fgf14^−/−^* mice is different from their male counterparts. This is especially critical given the gender differences in several domains of neuropsychiatric disorders, including the age of diagnosis, premorbid functioning, and symptom clustering [37,38,39,40,41]. These results indicate that there is a need to study behavioral changes, if any, in female *Fgf14^−/−^*. In fact, the upregulation of SNAP25 and MTATP6 not only indicate that female *Fgf14^−/−^* mice have a unique proteomic signature, but that they may have a mechanism of resilience that compensates for changes in the synaptic functions seen in male mice of the same age.

SNAP25 is an important member of the SNAP/SNARE complex, which is necessary for the proper release of vesicles at the synapse [85] and has previously been shown to co-immunoprecipitate with FGF14 [10]. Not only is the deletion of SNAP25 linked to an increase in E/I tone through increased glutamatergic neurotransmission [55], but the deletion of SNAP25 has also been linked to improper neurogenesis in the adult mammalian brain, which is an important endophenotype of several neuropsychiatric disorders, including SZ and bipolar disorder [86,87]. Previously, it has been shown that male *Fgf14^−/−^* mice also show traits of an immature DG [29]. These findings suggest that this could be mediated through SNAP25, although more research is needed to determine whether neurogenesis is also altered in the brain of adult female *Fgf14^−/−^* mice. Decreases in both SNAP25 and MTATP6 have been seen in patients with SZ, as well as in potential animal models of neuropsychiatric diseases [55,57,85,88,89]. Furthermore, genetic variants of SNAP25 leading to low protein expression levels have been associated with hyperactivity and/or with low cognitive scores in autistic patients [69,90], corroborating our results linking differentially expressed proteins in *Fgf14^−/−^* mice with autism (Figure 4). Not only does our study highlight the importance of sex-specific research in basic science, it lays the groundwork for further investigations on the mechanisms of potential resilience to neuropsychiatric disorders in females in preclinical models as well as in humans.

## Supplementary Materials

The following are available online at http://www.mdpi.com/2227-7382/7/1/5/s1, Figure S1: Male vs. Female WT volcano plot; Table S1: Protein fold changes, statistics, and proteomic results data from proteingroups.txt output from Maxquant. This file corresponds to actual data presented in Figure 3; Table S2: Autism and Schizophrenia associated genes, actual data for Figure 4; Table S3: Group-specific protein clusters identified from hierarchical clustering, complete protein lists used to prepare Figure 7.
